# Enriching for correct prediction of biological processes using a combination of diverse classifiers

**DOI:** 10.1186/1471-2105-12-189

**Published:** 2011-05-23

**Authors:** Daijin Ko, Brad Windle

**Affiliations:** 1 Department of Management Science and Statistics, School of Business, University of Texas at San Antonio, San Antonio, TX, USA; 2 UTSA Neuroscience Institute, University of Texas at San Antonio, San Antonio, TX, USA; 3 Department of Medicinal Chemistry, School of Pharmacy, Virginia Commonwealth University, Richmond, VA, USA; 4 Massey Cancer Center, Virginia Commonwealth University, Richmond, VA, USA

## Abstract

**Background:**

Machine learning models (classifiers) for classifying genes to biological processes each have their own unique characteristics in what genes can be classified and to what biological processes. No single learning model is qualitatively superior to any other model and overall precision for each model tends to be low. The classification results for each classifier can be complementary and synergistic suggesting the benefit of a combination of algorithms, but often the prediction probability outputs of various learning models are neither comparable nor compatible for combining. A means to compare outputs regardless of the model and data used and combine the results into an improved comprehensive model is needed.

**Results:**

Gene expression patterns from NCI's panel of 60 cell lines were used to train a Random Forest, a Support Vector Machine and a Neural Network model, plus two over-sampled models for classifying genes to biological processes. Each model produced unique characteristics in the classification results. We introduce the Precision Index measure (PIN) from the maximum posterior probability that allows assessing, comparing and combining multiple classifiers. The class specific precision measure (PIC) is introduced and used to select a subset of predictions across all classes and all classifiers with high precision. We developed a single classifier that combines the PINs from these five models in prediction and found that the PIN Combined Classifier (PINCom) significantly increased the number of correctly predicted genes over any single classifier. The PINCom applied to test genes that were not used in training also showed substantial improvement over any single model.

**Conclusions:**

This paper introduces novel and effective ways of assessing predictions by their precision and recall plus a method that combines several machine learning models and capitalizes on synergy and complementation in class selection, resulting in higher precision and recall. Different machine learning models yielded incongruent results each of which were successfully combined into one superior model using the PIN measure we developed. Validation of the boosted predictions for gene functions showed the genes to be accurately predicted.

## Background

The understanding of basic biological processes, diseases and drug actions depends on the discovery of the biological roles for genes. However, most human genes (~80%) are still not confidently annotated using Gene Ontology for biological process [http://www.ebi.ac.uk/GOA/]. Understanding gene function has recently been advanced by the use of machine learning models [1-10]. Models for classifying genes to biological processes, pathways or functional classes have been based on a variety of data from sequences and structures to gene expression under a variety of biological conditions [1,3-9]. Each model has yielded substantially different results from each other as a result of the different learning models used and the type of data. No one model can correctly classify genes for all biological processes, with each model having its own characteristics as to what biological processes classified well. Each learning method can identify patterns within the data that the others cannot, relevant to particular biological processes. The models show a complementarity as to what genes are classified and to what biological processes genes are classified, which points to the need to combine the best from all models [10].

Individually, the published classifiers have performed poorly overall with misclassification error rates of greater than 70%. Ko, Xu, and Windle [9] solved the high misclassification problem by controlling the error rate using a class-by-class filtering procedure to increase precision, however, this approach sustained a reduction in the number of genes classifiable. Combinations of learning models and data have been shown to greatly improve the confidence in gene classification and increase the number of genes classifiable at high confidence [10,11]. However, the combining of models or model ensembles have thus far relied on using similar learning models that yield similar outputs [11-13]. Combination of models from substantially different learning algorithms suffers from the problem that the model outputs (estimated posterior probabilities) are too dissimilar, providing no unified measure for assessing the models for comparison or the framework for combining models in an equitable manner.

In this paper, we have investigated the diversity in performance and output on a class-by-class basis for a collection of different machine learning models using a set of gene expression data. Each resultant classifier exhibited a different performance with unique outputs for each biological process class. We developed a unifying measure, Precision index (PIN), a transformation of the maximum posterior probability as a measure of prediction accuracy that allowed us to not only rank the prediction precision from each model but also provide prediction error at any precision point. The PIN measure was used in combining the different models into a modeling process that capitalized on the synergy and complementation within models. This PIN Combined Classifier (PINCom) resulted in higher number of correct predictions than any single model or method while maintaining high prediction accuracy. The class specific precision measure PIC was used in filtering predictions across all models to produce a large set of predicted genes with a desired precision. The results are a substantial improvement in both precision and recall for classifying genes to functions.

## Methods

### Combining classifiers

The task of a classifier is to "learn" from the examples in which we know to which classes the observations belong and predict the class for future observations. One can show that under some assumptions, the classification rule "Classify x into the class with highest posterior probability p(c|x)" minimizes the total risk in a sense [14]. Therefore, performance of a classifier will be dependent upon whether the classifier provides a good estimate of the posterior probabilities of this class membership (p(c|x)). Most classifiers provide some sort of estimate of such probabilities but they may not represent probabilities in a strictly stochastic sense. In this paper, we follow the notation used in standard machine learning textbooks [14-16] and use the terminology "posterior probabilities of class membership (p(c|x))" somewhat loosely.

Define maximum posterior probability MaxP(x) of x, as max_j _p(j|x), the maximum value of the class membership probability. The MaxP(x) is an index that may be used as an indication of prediction accuracy. For example, if we were to choose a subset of highly confident predictions, we could select predictions whose MaxP is large. In general, if the classifier is good, we expect that the higher the MaxP, the more reliable the prediction. Therefore, along with the predicted class, the corresponding maximum posterior probability MaxP could be used to rank the prediction precision.

Though MaxP could be used as a measure of prediction accuracy, it is not a probability measure in any sense and therefore a MaxP from one classifier cannot be compared to the MaxP of another classifier unless they belong to the same family of classifiers. Consequently, one cannot say the prediction from one classifier is better than the prediction from other classifier based on the order of the corresponding MaxP.

In this section, we develop a unifying measure, Precision index (PIN), as a 'probabilistic' measure of prediction accuracy, which allowed us not only to rank the prediction precisions within each classifier but also to rank the prediction precisions among different classifiers and develop a 'PIN Combined Classifier', which combines and makes a decision rule based on PIN measures.

Following the information retrieval theory, we define the precision of a set A of predictions as the proportion of correctly predicted elements in A while recall is defined as the proportion of the correctly predicted element in A in the whole data space. Consider {(_f_y_i, _y_i _, x_i_, MaxP(x_i_)), i = 1,..., n} to be the set of predictions from a classifier where _f_y_i _is the predicted class for the input data vector x_i_, y_i _is the true class, and MaxP(x_i_) is as defined above. We define Pindex(a) as the Precision of the set A = {x | MaxP(x) ≥ a}, for 0 ≤ a ≤ 1. The Pindex(a) is estimated by the number of correctly predicted elements in the subset {(_f_y_i, _y_i _, x_i_)| MaxP(x_i_) ≥ a} divided by the number of elements in the set A.

The function Pindex( ) for a classifier is discrete function and could be assumed to be monotonically non-decreasing. This assumption is based on our belief that the higher the MaxP, the better confidence we have in the predictions. For some classifiers, the Pindex values of each predicted data point could be non-monotonic on some area of MaxP, in which case we are not able to use MaxP as a discriminant value of goodness of the prediction and Pindex estimated under the monotonicity assumption would be constant on the area of such MaxP.

The observed Pindex(x_i_) of the data point x_i _is defined as Pindex(MaxP(x_i_)). We estimate a refined monotonically non-decreasing Precision index function by fitting an isotonic regression of the data {(MaxP(x_i_), Pindex(x_i_)), i = 1,..., n}. In this paper, we used an isotonic regression [17] and linear interpolation for the estimation. We will use the notation PIN() (Precision Index) as the refined monotone non-decreasing Precision index function and the notation PIN(x_i_) for PIN(MaxP(x_i_)) for convenience.

Unlike the MaxP, PIN is a comparable quantity among the different classifiers and can be compared to PIN values from other classifiers. Let M_1_, ..., and M_L _be a set of classifiers and Pin^M1^(x), Pin^M2 ^(x)_, _..., and Pin^ML^(x) be the corresponding Precision indices (PINs) of a point x, and y^M1^(x), ..., y^ML^(x) be the corresponding predicted classes. We define a new classification rule "Classify x into the class with the highest PIN value" and call it the PIN Combined Classifier (PINCom) or the Combined Classifier. The PINCom has the predicted class y^c^(x), and the measure max_Mi _Pin^Mi^(x) is named as MaxPIN. The measure MaxPIN plays the role of MaxP for the PINCom.

## Recall-Precision curve

Precision P(a) of a subset {x | PIN(x) ≥ a} is estimated as the number of correct predictions divided by the size of the subset and recall R(a) is estimated by the number of correct predictions in the subset divided by the size of the whole set of predictions. The set of points {(P(a), R(a))| 0≤ a ≤ 1} is called the Recall-Precision curve and used in assessing classifiers. The P(0) and R(0) are the overall precision and recall of the whole sample space and therefore they are of equal value. The primary goal of a classifier is to have a high P(0) value and the comparison of classifiers is often made by comparing P(0)'s (or R(0)'s) of the classifiers. In this paper, we are more concerned about selecting a set of predictions with high precision and want to find the classifier that generates the largest of such set. Ideally, it would be a classifier that dominates the whole Recall-Precision curve instead of the overall precision and recall alone.

The recall R(a) measures the fraction of elements in the subset {x | PIN(x) ≥ a} that are predicted correctly in the whole prediction space and measures the productivity of the selected predictions of the classifier. The precision P(a) measures the fraction of correct predictions in the given set {x | PIN(x) ≥ a}. For a fixed precision P(a), we can find a classifier that maximizes the recall R(a) among the classifiers and for a fixed recall, we can find a classifier that maximizes the corresponding precision. The task of obtaining the largest set of predictions with fixed precision or the set of highest precision with a fixed recall can be accomplished by selecting the 'optimal' classifier and filtering predictions via its PIN values.

Recall-Precision curves for a set of classifiers are used to assess the classifiers' performance. For a fixed precision value, say P_0_, each R value that intersects with the vertical line P = P_0 _measures how many predictions are correctly predicted from the corresponding classifier. Similarly for a fixed R value, say R_0 _, each P value that intersects with the horizontal line R = R_0 _measures the precisions of the corresponding subset of predictions that yields the fixed R_0 _amount of correct predictions. When a classifier is not efficient in distinguishing the good predictions from the bad predictions, R(a) and P(a) becomes constants for all 'a' and the generated Recall-Precision curve would become a single point.

The precision curve P(a) is used in finding threshold value a_0 _and the corresponding subset of predictions that reach the given prediction P_0 _by solving the equation P(a_0_) = P_0 _or (a_0 _= P^-1 ^(P_0_) where P^-1 ^is the inverse function of P). Such subset has the recall R(a_0_). For the classifiers M_1_, ..., and M_L _, let P_M1 _,..., R_ML _and R_M1 _,..., R_ML _be the corresponding precisions and recalls. The threshold of PIN values for the subset that maintains precision P_0 _for classifier Mi is a_Mi _= P_Mi_^-1 ^(P_0_) and the corresponding R value for the classifier Mi is R_Mi_(a_Mi_). Similarly a subset of predictions whose recall is R_0 _can be obtained by the threshold value a_Mi _= R_Mi_^-1 ^(R_0_). The corresponding precision of the subset is P(a_Mi_).

For a fixed precision P_0_= P^Mi^(a_Mi _), the corresponding R^Mi^(a_Mi_) can be compared and the best classifier is chosen as the one that maximizes R^Mi^(a_Mi_). Equivalently, when an R value is fixed, the desired % of correct predictions out of the whole prediction set is fixed so the P^Mi^(a_Mi_) values of the corresponding sets are the precisions of the selected sets from classifiers and the best classifier is the one that maximizes P^Mi^(a_Mj_). A comprehensive assessment of the classifiers could be made by comparing the whole Recall-Precision curves.

For the PIN Combined Classifier, we use MaxPIN in constructing the Recall-Precision curve, i.e., P_C_(a) is the precision of the subset {x | MaxPIN(x) ≥ a} of predictions and R_C_(a) is the recall of the subset. The P_C _and R_C _functions are estimated by the counting the number of correct predictions in the set of predictions. For notational simplicity, we use PIN_C _for MaxPIN. For previously stated reasons, we assume that the precision curve P_C_() is a non-decreasing function and recall curve R_C_() is a non-increasing function. As previously stated, they are estimated by fitting a monotone increasing ("isotonic") function (an isotonic regression [17]) and linear interpolation for P_C _and monotone decreasing ("antitonic") function and linear interpolation for R_C_.

## Class-specific Recall-Precision curve

Though the overall Recall-Precision curve is used in selecting the 'best' subset with a fixed precision, the maximum precision of a classifier may not reach the desired precision level. This is often the case when the classifier and the corresponding precision index used in thresholding are ineffective in distinguishing good predictions from bad ones. When the training data itself is noisy, it is hard to build an effective classifier and hence the maximum precision would only reach a moderately large value. Though a classifier may not effectively classify elements in all classes, it may classify elements in some classes very well. In such a case, one could select the predictions in the effectively classified classes [9].

In the following, we introduce the class specific Recall-Precision curve that estimates precision and recall in the set of predictions whose predicted classes are fixed. For some classes, the precision curve could be worse than the overall precision curve (reaching smaller values) but for some classes, the precision curve could reach much higher precision. We use this class specific Recall-Precision curve to find a subset with a desired higher precision. Precision P^Mi ^_k_(a) of a given class k for the classifier M_i _is defined as the precision of the set {x | PIN(x) ≥ a and y^Mi^(x) = k} where y^Mi^(x) is the predicted class of x by the classifier M_i_. It is estimated by the number of correct predictions within the predicted class k divided by the number of predictions whose predicted class is k. The recall R^Mi ^_k_(a) is estimated by the number of correct predictions in the subset divided by the size of the set of all predictions whose true class is k. For the data point x and the classifier M_i _whose predicted class is k, we define PIC(x), the Class Specific Precision Index (or Precision index in Class), as P^Mi ^_k_(PIN(x)). The PIC values often spread more and reach higher values than overall PIN values and enables us to select a set of predictions with a high precision. We can use PIC in construction of Recall-Precision curve as we use PIN. The resulting curves will enable us to compare the class specific productivity of different classifiers.

## Construction of Combined Classifier for test data

When several classifiers, say M_1_, ..., and M_L _are applied to a test data, the classifier PINCom is constructed as follows.

### Step A1: Estimation of PIN in training data

1. Partition randomly the training data into 10 subsets.

2. Train the classifiers using 90% data and apply them to the 10% for each partitioned data.

3. Repeating the procedure 10 times generates cross-validated predictions of size of training data.

4. Repeat 1 - 3 for K random partitions where K is around 20-50.

5. The generated predictions are of size K times of the training data size for each model. The predicted points are not independent because they are generated by the same training data.

6. Estimate the PIN function for each model based on the predictions, predicted classes, and the true classes.

7. PINCom's predicted class of a point is the predicted class of the classifier with maximum PIN value.

### Step A2: Estimation of PIN in test data

1. Train the classifiers on the training data and apply them to the test data.

2. Each prediction from the model M_i _consists of (x_j_, y^Mi^(x_j_), MaxP(x_j_)) for j = 1, ..., n_T _where n_T _is the size of the test data set.

3. Apply the estimated PIN function from step A1 to the test data predictions and get PIN values for the test data prediction, i.e., PIN(x_j_) = PIN(MaxP(x_j_)).

4. The prediction of the PINCom is the predicted class of the classifier that has the largest PIN value. When more than one classifier's PINs are of maximum PIN value, select one classifier randomly. We use MaxPIN as the PIN_C_, the PIN value of the PINCom.

## Evaluation of classifiers

We evaluate the effectiveness of classifiers on test data by Recall-Precision curve plots. When the true classes of the test data are known, we estimate the Recall-Precision curves based on PIN and PIN_C _values estimated in step A2. They are based on estimated PIN functions in step A1 that are based on the cross-validated prediction on training data. The Recall-Precision functions are based on the predicted outcome class, PIN values and true class in step A2. The class specific Recall-Precision curves PIC can be used in evaluating class-by-class predictions. PIC based subset selection and Recall-Precision curves are used in comparing classifiers' class specific recalls with preset precision level. The true classes of test data are usually unknown. In this case, we evaluate the classifiers on training set by double cross-validated predictions as follows.

### Step B1

1. Partition the Training Data into 10 subsets.

2. Allocate each partitioned data (10%) as test set and use remaining 90% set as a new training set as in Step A1.

3. Partition the new training set into another 10 subsets and generate 10-fold cross-validated predictions for each model. In each cross-validated prediction, 81% of the original training data is used in training classifiers and 9% in generating cross-validated predictions. In the end, a set of cross-validated predictions of the size of the new training data is obtained.

4. Repeat the above procedure for K random partitions (in our study we use K = 20) to generate more cross-validated predictions. We will have a cross-validated prediction set of size 20 times of 90% of the original training data.

5. Estimate the PIN function for each classifier using the cross-validated prediction set in 4.

6. Generate Combined Classifier predictions based on PIN and MaxPIN.

7. The generation of PIN, MaxPIN, and therefore predictions of the PINCom are based on the predicted values and the true class levels of the new training set (90% of data) in 2.

### Step B2

1. Train the classifier on training set (90% data) in step B1.1 and apply it to the test set and generate predictions of 10% of the data.

2. Apply the PIN functions generated by the data in step B1.3-B1.7 to the test data predictions.

3. Generate the Combined Classifier predictions based on the estimated PINs and MaxPIN in B2.2.

4. Repeat 1-3 for all 10 partitioned test sets and have cross-validated predictions of the size of the original data.

5. Repeat 1-4 for KK random partitions. We use KK = 10 in this paper.

6. We have generated double cross-validated predictions for the all the classifiers including the classifier PINCom.

7. Each prediction consists of the values PIN for each classifier, MaxPIN (or PIN_C_) for the PINCom Classifier, predicted class for each classifier, predicted class for the classifier PINCom, and the true class.

8. Generate Recall-Precision curves and PIC values based on the set of double cross-validated predictions generated from B2.1-B2.7.

Note that the PIN, PIN_C _and the PINCom are based on the training data only and hence the performance of the classifiers based on the double cross-validated predictions provide valid evaluation for the PINCom as well as the other classifiers used in combining. The need of double cross-validation is discussed in detail in [18] for example.

We applied the classifiers to the test data set whose true classes are unknown as follows.

1. Build classifiers based on all the training data and apply them to the test data set.

2. Estimate PIN, PIN_C_, and the PINCom classifier predictions using double cross-validated predictions described in steps B1 and B2.

3. For each classifier including the PINCom, estimate the functional relationship between PIN and PIC, the class specific precision from the double cross-validated training data as in step B2.8 and apply it to the test data.

4. The subset {x| PIN(x) ≥ a } of the test data has the estimated precision P(a) of the subset {x| PIN(x) ≥ a } from the training data. To avoid the confusion, we use the notation P_xv_(a) for the precision from the double cross-validated predictions from the training data.

5. The estimated number of correctly predicted genes in a set of predictions in test data is the size of the subset multiplied by P_xv _(a) and hence the P(a) and R(a) of the test data can be estimated by dividing the estimated number of correctly predicted genes by the subset size and the test data size, respectively.

6. Appling the same arguments, the class specific precision P_k_(a) from the double cross-validated training predictions can be used in estimating the number of correct predictions. For classifier Mi, the number of correctly predicted predictions in predicted class k is estimated by the number of elements in {x| PIC(x) ≥ a and y^Mi^(x) = k} multiplied by P_K_(a), the class specific precision.

7. The number of correct predictions of the set {x| PIC(x) ≥ a } is estimated by adding all the estimated class-specific correct predictions.

8. The precision P(a) of a subset {x| PIC(x) ≥ a } of the predictions of test data is estimated by dividing the estimated correct number of predictions by the subset size.

9. The overall recall R(a) is estimated by summing the numbers of correctly predicted elements in each predicted class divided the total predicted elements.

## Classifiers and software

The neural network (NN) classifier was developed using nnet library in R [14,16], and the training data described in the Results section. NN classifiers are based on a random number generator and may contain local maxima. We averaged 10 NN runs (each with a different random number generator seed) and averaged the posterior probability vectors to produce a stable prediction as recommended in [14,16]. The optimal NN parameters were chosen to minimize the 10 fold cross-validated prediction errors. The random forest (RF) model [12] was developed using the randomForest function in randomForest library in R [13]. The multi-class support vector machine (SVM) model [19,20] with the radial kernel function was developed using the svm function in the library e1071 [21-23] in R. The radial kernel function was used and optimal parameters were chosen to minimize 10-fold cross-validated prediction error.

In addition to the original data, we built the over-sampled training data by duplicating small size classes to the largest class size. When a classifier is applied to the oversampled training data, we call it the oversampled version of the classifier. The over-sampled versions of RF and SVM were used because some classes used in training have fewer genes than others and without equal weight, might not classify well. Therefore, over-sampling was used to increase the weight of smaller classes, resulting in better classification for those smaller classes as well the bigger classes. Classification to the bigger classes is advantageous because the number of predictions from the contaminating incorrect genes from the smaller classes goes down. In our cross-validated study, over-sampling indeed produced better results on the accuracy in some classes. When the oversampled versions of classifiers are added in combining classifiers the improvement of precision and recall was noticeable. R-scripts are available from the authors to implement all methods described.

### Other methods of combining classifiers

The vote-combining method (Vote) uses the most frequently voted class among the classifiers. Ties were broken by random selection. This method has been shown to greatly improve the confidence of the predictions but thus far mainly relied on vote-combining of similar learning models that yield similar outputs [11-13]. This method could be adversely affected by low performing classifiers when substantially different classifiers are combined.

Stacking or Stacked generalization [24-26] is another method for combining classifiers that overcomes these problems by taking weighted average of output. In Stacking, the weights are determined by a higher-level learning classifier and are expected to be proportional to the capability of the classifiers used. In spirit, Stacking is similar to our PIN Combined Classifier. Both use the outcome of the lower level classifiers and use double cross-validation to assess the accuracy of the prediction. We studied two stacking methods in addition to the vote combining method and compared them with the PINCom. The two stacking methods we used were as follows.

The first step of stacking is to collect the output of each model (level-0 classifier) into a new set of data (level-1 data). For each instance in the original training set (level-0 data), this data set represents every model's prediction of that instance's class, along with its true class. This is achieved by ordinary cross-validation. The new data is treated as the data for another learning problem, and in the second step, a learning algorithm (level-1 classifier or meta-classifier) is employed to solve this problem.

The Stack 1 method uses the output class probabilities generated by level-0 classifiers to form level-1 data. Then, as the level-1 classifier we use a version of least squares linear regression adapted for classification tasks, called the multi-response linear regression (MLR), which adapts each class outcome as 0-1 outcome variable in regression. Since the coefficients of the regression would be expected to be higher values for the better classifier's output class probabilities, the resulting procedure would have an improved predictive accuracy compared to the level-0 classifiers. The second stacking method, Stack 2, uses the output class predictions as well as class probabilities generated by level-0 classifiers to form level-1 data. Then, a Random Forest is used as the level-1 classifier. Both stacking methods are evaluated at the test data set for their prediction ability and when no test set is available, a double cross-validation is used in evaluating as we do in evaluation of the PINCom.

## Results

### Data set and classifiers

We explored ways to best classify genes to biological processes using gene expression data. We used the gene expression data used in the neural network model study by Ko et al. [9]. The data are originally from the study by Ross et al. [4]. Sixty human cancer cell lines from 9 different cell types exhibit varying levels of gene expression that are heavily based on biological processes in each particular cell line. The varying levels of gene expression in the 60 cell lines represent a multiplex of activities for pathways and other biological processes, which have been proved very useful in classifying genes to biological processes. The 21 gene functional classes chosen from the KEGG database were assigned to 367 genes chosen from 6165 genes profiled for gene expression in the 60 cell lines. The class size for biological processes ranged from 8 to 50.

Various modeling algorithms can each identify different patterns within data that can be useful in classification of different gene functions. Therefore, we investigated multiple classifiers for the ability to classify genes to functions with a focus on each biological process class. We selected 3 classifiers plus a variation of two of these classifiers that performed well individually for classifying genes to biological processes; these are random forest (RF)[12,13], multi-class support vector machine (SVM)[19,20], and neural network (NN)[14,16], plus over-sampled versions of RF and SVM (RFO and SVMO). These classifiers were selected based on their ability to classify well, as well as being able to generate output results in the form of estimated posterior probabilities for all classes of biological process. We investigated the performance for each classifier on a class-by-class basis for the 21 biological processes.

In comparing the performances for each classifier, a problem exists in which the distributions of maximum posterior probabilities (MaxPs) are vastly different from class to class [9] and classifier to classifier, particularly when the learning model algorithms are vastly different. The MaxP of an element from one classifier cannot be directly compared to the MaxP of the same element from another classifier and one cannot discriminate one classifier from another by the MaxPs.

### Comparison of classifiers for classifying genes to biological processes

**Figure 1:**
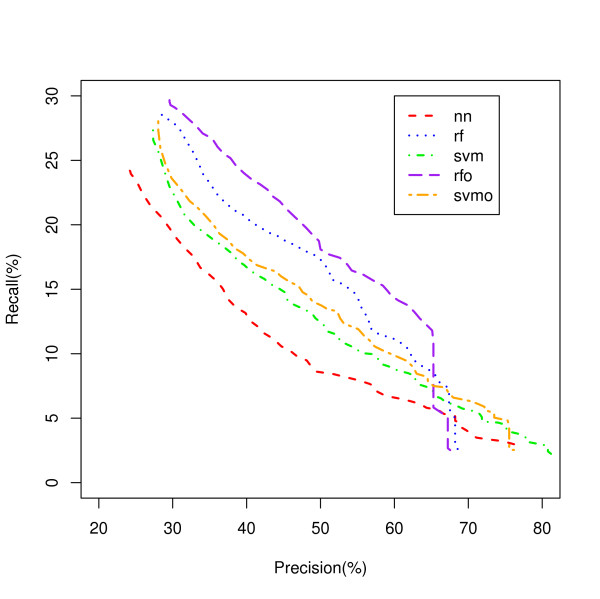
**Overall Classification of Genes to Biological Processes**. Five classifiers classifying genes to all biological process classes are compared using a Recall-Precision curve. rf - random forest; rfo - random forest oversampled; svm - support vector machines; svmo - support vector machines oversampled; nn - neural network.

We addressed the classifier comparison problem by developing a measure of performance and prediction confidence, referred to as Precision Index (PIN) that allows us to directly compare results between classes and between classifiers (see Methods). We transformed MaxPs for each classifier to PINs based on precisions within each classifier, which allowed us to compare the precisions of all classifiers. We filtered the predictions based on PIN values, calculating a precision for the set of selected predictions that has greater than each PIN threshold. We evaluated recall, the fraction of genes attributed to a particular biological process that are correctly predicted, for each class and each model for the set of selected predictions that has greater than each PIN threshold. For various thresholds, we are selecting predictions that have PIN values of at least the threshold and evaluating the recall of the set of selected predictions and its precision, which provides a measure of how good the classifier is in selecting predictions with a given precision (see Methods). A Recall-Precision curve of the set with each PIN threshold is shown in Figure 1 allowing us to see what classifiers perform the best for biological process classifications overall.

Figure 1 shows the RFO classifier has superior recall to the other models for sets of lower precision, while the SVM classifier has superior recall for sets of higher precision. However, analysis of each class for biological process revealed dramatic differences between classifiers dependent on the class.

**Figure 2:**
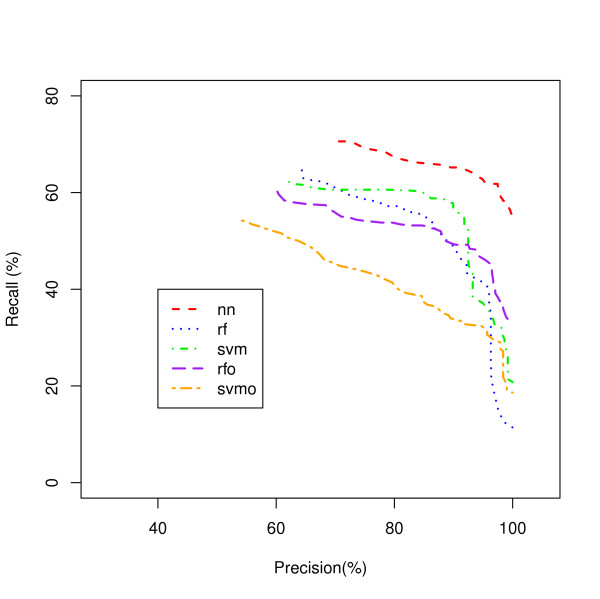
**Ribosome Process Classification**. A comparison of five classifiers for the Ribosome protein genes.

**Figure 3:**
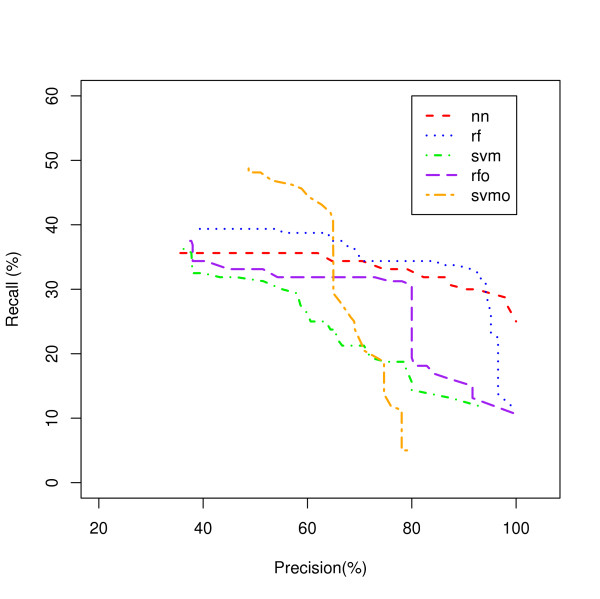
**Proteasome Process Classification**. A comparison of five model classifiers for the Proteasome genes.

A Recall-Precision curve plot is shown in Figure 2 for the Ribosome class as an example. The NN model, the worst classifier in Figure 1 showed the best recall over a wide range of precisions followed by the RFO and SVM. Another example is shown for the Proteasome class in Figure 3. In this class, SVMO is dominating at the lower precision values and RF is superior for the precision from 65% to 95% while NN is dominating on very high precision area (95% and up). Our analysis shows that it is difficult to identify a single classifier that dominates in recall value for all precisions and all classes.

### Combining classifiers based on PIN

**Figure 4:**
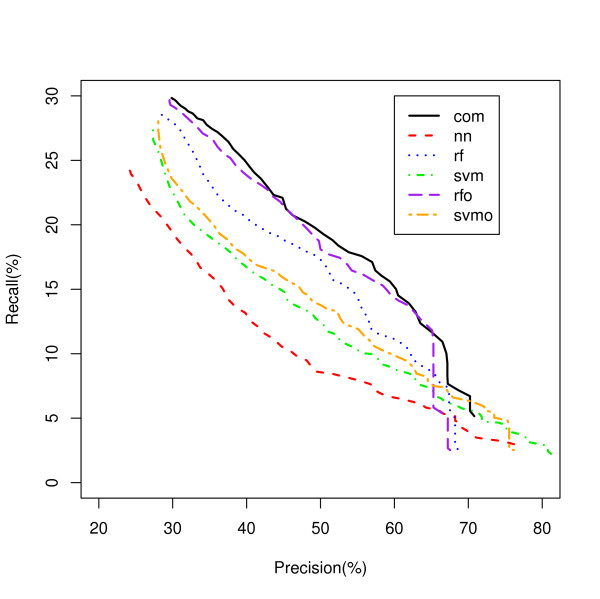
**Overall Gene Classification by the PIN Combined Classifier**. Five classifiers classifying genes to all biological process classes are compared to the PIN Combined Classifier. com - PIN Combined Classifier (PINCom).

Considering all classifiers for which there is no single dominant classifier under any one condition, we use a new classifier PINCom (Methods). This method is different from the classifier ensemble methods. In the classifiers ensemble methods, one combines many classifiers with similar characters (classification trees or support vector machines) and uses weighted voting based on predictions and estimated posterior probability [10,12]. In PINCom, we combined classifiers whose characteristics could be quite different from one another in which their estimated posterior probabilities were not comparable. The development of PIN allows us to compare and combine the five different classifiers we developed. The maximum PIN defines the assigned classification of the Combined Classifier and it was compared to the five other classifiers using a Recall-Precision plot (Figure 4). These results show a clear advantage in classification recall for the PINCom over all other models for precision areas up to 70% closely following the performance of the best model but dominating it at precision values greater than 65%. However, the PINCom's precision reaches only up to 70%, meaning if we wanted to select a set of predictions with precision of say 95%, we would be unable to use it.

**Figure 5:**
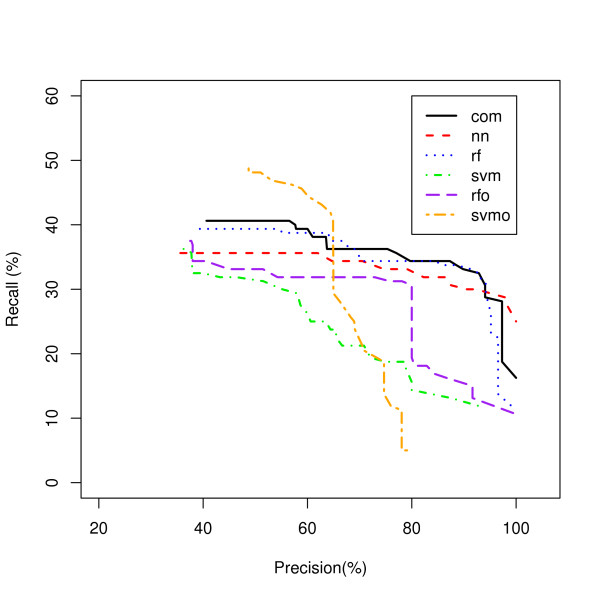
**Ribosome Process Classification by the PIN Combined Classifier**. Five modeling methods classifying genes to the Ribosome class are compared to the PIN Combined Classifier (PINCom).

**Figure 6:**
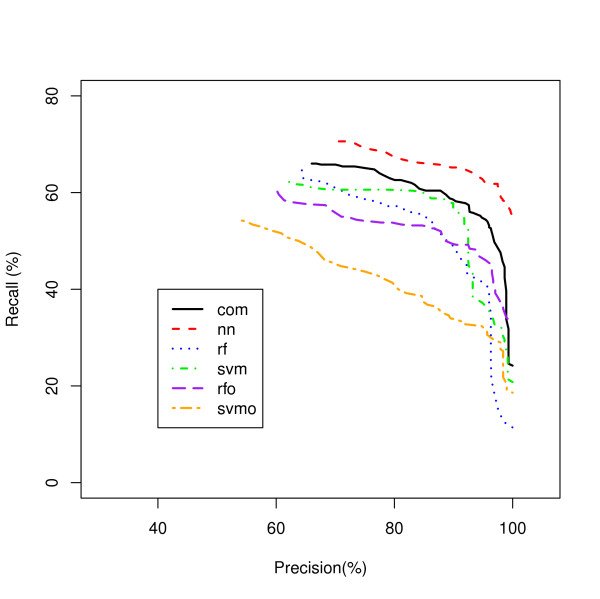
**Proteasome Process Classification by the PIN Combined Classifier**. Five modeling methods classifying genes to the Proteasome class are compared to the PIN Combined Classifier.

Figures 5 and 6 show the Recall-Precision curves for the PINCom for the Ribosome and Proteasome classes already described in Figures 2 and 3. The Combined Classifier was superior to all individual classifiers in Proteasome class and in Ribosome class to all others but the neural network classifier. It is important to note that even if the overall precision of the Combined Classifier could reach only up to 70%, the class specific precision could reach up to 100%.

**Figure 7:**
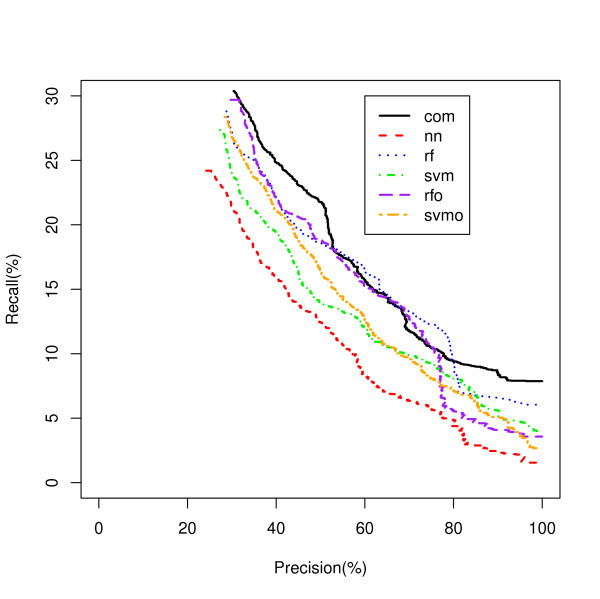
**Recall-Precision curve based on PIC**. Recall-Precision curves based on the class-specific Precision index (PIC) of 5 classifiers are compared to the PIN Combined Classifier (PINCom).

We analyzed the class specific precision index PIC (Methods) based on PIN values in each class and found we could reach higher precisions. Figure 7 shows the Recall-Precision curves based on PIC thresholds. It can also be generated by a weighted average of class-specific Recall-Precision curves with weights proportional to the sizes of the predicted classes. The recalls of the PINCom are dominating particularly at the high precision levels and at most of the precision levels. The precision limit is thus extended to a much higher value (100%).

### Application of the PINCom to the test data with unknown true classes

We applied the PINCom to the test data set of 5798 genes that were not used in model training. In building the PINCom for the model, we used the PIN functions, the relationship between MaxP and Precision Index PIN, estimated by predictions from 10-fold cross-validated predictions from our training data with 367 genes. We used 10 random partitions and generated cross-validated predictions of size 3670 in estimating PIN function and applied it to the test data. The PIN values of the five models in the cross-validated prediction are used in building the PINCom. The functional relationship between MaxP and PIN is applied to the MaxP of the test data resulting in PINs for the five models and consequently the PINCom for the test data.

**Figure 8:**
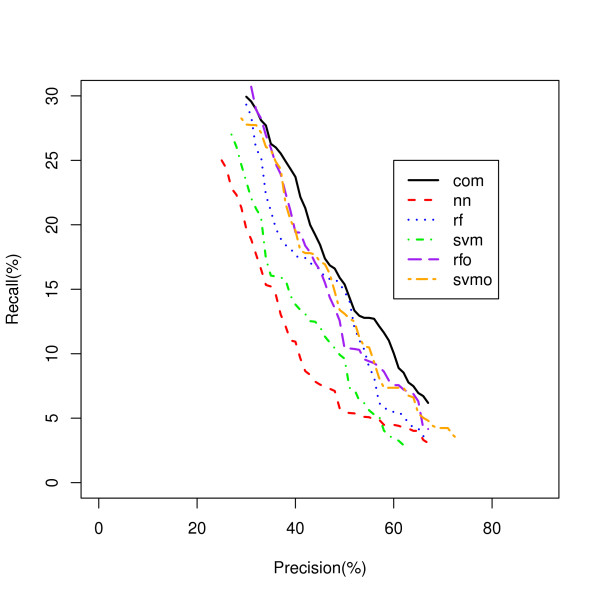
**Combined Classifier Performance on Test Data**. The five classifiers and the PIN Combined Classifier (PINCom) are applied to the test data for overall gene classification evaluated by a Recall-Precision curve.

Figures 8 shows a Recall-Precision plot for the test data. Since we do not know the exact class to which these elements should belong, the number of correct predictions is estimated using the precision estimate from the double cross-validated training data (Methods). The precision and recall are estimated by the precision from the training data, hence the PR curves in Figure 8 assume that the training data represents the test data. We observed that the PINCom was vastly superior to all other models at most of the precision levels up to 70%.

**Figure 9:**
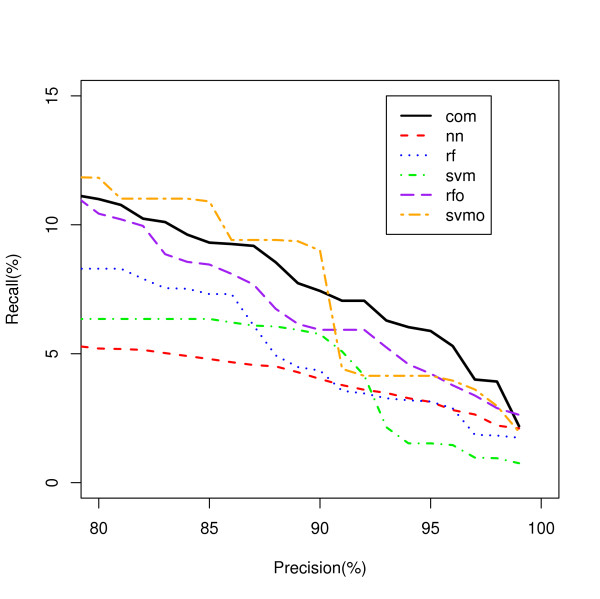
**PIN Combined Classifier Performance based on PIC on Test Data**. Application of the five classifiers and the PIN Combined Classifier (PINCom) using test data for overall gene classification evaluated by Recall-Precision curves based on the class-specific Precision index (PIC).

We would like to select the predictions with much higher precision than 70%. We estimated the functional relationship between PIN and PIC in each class for each classifier from the double cross-validated predictions from the training data and applied it to test data to estimate the PIN and PIC values as well as the corresponding precision and recall (Methods). Because we used PIC in selecting predictions with high precision, we compared the Recall-Precision curves only on the higher PIC value regions shown in Figure 9. PINCom dominates all the individual classifiers at the most of high precision levels in Figure 9. The SVMO performs slightly better than PINCom at the precision levels from 80% to 90% region but much worse than PINCom in the 90% and up regions.

### Test validation of Combined Classifier predictions

When the PINCom was applied to the 5798 test genes, we expected that genes predicted for a biological process would include genes that have known associations with that process based on data from other studies as determined by other gene annotations, such as Gene Ontology (GO). Our test-validation results showed that the Cell Cycle pathway showed a substantial improvement for predictions by the PINCom over individual models. At a PIC of 1.0, there were 54 genes predicted for Cell Cycle by the PINCom yet zero genes predicted by any individual model, which is a performance comparable to what we observed in the cross-validation. We analyzed the 54 genes for associations with genes within gene annotation databases using the DAVID Functional Annotation Tool [27]. Within the 54 predicted genes, there were 14 genes intersecting with the UniProt biological process for Cell Cycle with a p-value = 4.9 × 10^-11^, and an FDR of 5.8 × 10^-8^. This verifies that our PINCom is correctly predicting genes for Cell Cycle even under conditions in which the individual models yielded no genes, thus the additional genes classified by the PINCom at high confidence represent a legitimate improvement in predictions.

An interesting application of our Combined Classifier is to look at gene predictions that appear contrary to what is believed to be true or that provide new information. We analyzed genes predicted for Cell Adhesion from PINCom for associations with gene annotations using the DAVID Functional Annotation Tool. We found a statistically significant association with the GO biological process of Cell substrate adhesion with a p-value of 2.4 × 10^-6 ^and an FDR of = 3.7 × 10^-3^. This was consistent with the function of Cell Adhesion. We also observed a significant association with 5 genes from UniProt Metal Binding (p-value of 2.7 × 10^-6^, FDR of = 3.5 × 10^-3^). This category represents metallothionein genes. These results reveal a pattern of expression for these metallothionein genes that is similar to Cell Adhesion genes, and thus accounting for the classification. However, published studies only show a role for metallothioneins in metal transport and storage, and as well as response to oxidative stress [2]. No role in Cell Adhesion or related functions has yet been established experimentally for metallothioneins.

### Contribution of individual models in the Combined Classifier

We evaluated the contribution of each classifier to the Combined Classifier by determining the difference in recall (%) between the Combined Classifier based on 5 models for each precision and the Combined Classifier based on 4 models, withholding the classifier of interest. A minus difference means that the Combined Classifier without 1 classifier performs better that the 5 classifiers combination. The results summarized in Table 1 shows the NN classifier was most positively influential for most of the precision values and the SVM model was most positively influential at the higher precision values. Each classifier contributed positively at most of precision values, though, the RF model had the smallest contribution overall. One reason for this could be that the contribution of RF is compensated by a similar model, RFO.

**Table 1:** Contribution of individual models in the Combined Classifier (%)

Precision (%)	nn	rf	svm	rfo	svmo
**30**	8.58	2.17	4.24	0.40	3.21
**40**	7.37	3.64	4.64	3.76	2.78
**50**	4.87	2.88	3.27	4.62	2.62
**60**	5.87	2.23	3.20	4.92	3.14
**70**	1.39	-0.07	6.29	0.64	0.32

## Comparison of PINCom to other combining methods

We used two other methods for combining classifiers, voting and stacking as described in Methods, for comparison to PINCom. The voting method, Vote, uses the votes for classification from the individual classifiers. Stacking uses the output from individual classifiers to train a higher level classifier [25-27]. We used two versions of stacking, Stack 1 and Stack 2 (Methods).

Table 2 shows the recalls of Vote and Stack 1 and Stack 2 classifiers in comparison to the PINCom classifier at the various precision levels using the gene expression data. In this data, the overall precision of Vote is 29.7% beating all the other classifiers except the PINCom. The Vote suffers a great deal at the precision levels of 40%, 50% and 60% with recalls lower than other methods. Though the overall precision is comparable to the best classifiers, its use in selecting high precision predictions is not recommended.

**Table 2:** Recall Percentage of the Combined Classifiers at different precision levels for 60 Cancer Cell Line Data

Precision	Overall (%)	40%	50%	60%	70%
**Vote**	29.7	21.5	14.3	0	0
**Stack 1**	22.1	14.7	12.5	10.9	0
**Stack 2**	26.7	19.1	15.5	12.8	8.4
**PINCom**	29.8	24.7	19.4	14.3	6.1

The overall precision of Stack 1 is 22.1%, lower than Vote and even lower than level-0 classifiers. The recalls for Stack 1 at the given precision levels are also very low. The reason of this performance of Stack 1 could be due to large number of classes and relatively small size of data. There are 21 classes and hence level-one data have 100 independent input variables. With about 300 data from the cross-validated training, the level-1 classifier MLR might have over-fitted the training data.

The overall precision of Stack 2 is 26.7% lower than some of the level-0 classifiers. The recalls at the given precision levels were better than some of level-0 classifiers and Vote. The PINCom has the overall precision 29.8, with recalls higher than the other combining methods, thus demonstrates its superiority to existing combining methods.

### Application to the 'Vowel Recognition' Data

We used a well-known 'Vowel Recognition' data set to verify that the proposed classifier PINCom performed better than other classifiers used in the combining and other combining methods described above. The 'Vowel Recognition Data' were collected by Deterding [15], who recorded examples of the eleven steady state vowels of English spoken by fifteen speakers for a speaker normalization study. There are 528 training observations and 462 test observations consisting of 11 classes and 10 predictor variables. The data have been analyzed in various methods and are reported in a popular textbook [15, page 396] and can be downloaded from the website http://www-stat-class.stanford.edu/~tibs/ElemStatLearn/.

We used 5 different classifiers, random forest (rf), neural network with one hidden layer of 10 units (nn), support vector machine with radial kernel (svm), k nearest neighbor [15] for k = 6 (knn), and multivariate adaptive regression spline [15](mars) with 15 maximum number of terms and the PINCom (com) of 5 classifiers. The outcome variable (vowels) in Vowel training data is balanced (each class has 48 training data points) and oversampled versions of rf and svm we used previously did not provide additional benefits to the original rf and svm. Therefore, we replaced the oversampled models with two additional classifiers, knn and mars.

The optimal parameters were selected in cross-validated training set except the knn. The optimal k for knn was 1, which resulted in constant maximum prediction probability of 1. Therefore, the Precision-Recall curve became a single point and hence could not be used in distinguishing good predictions from the bad predictions. To introduce some variability of prediction probability we chose 'optimal' k among the values greater than 5 with the optimal k = 6.

We applied the trained classifiers from the whole training data set to the test data and estimated PIN function from the cross-validated predictions and MaxP. The trained classifiers on the training data were applied to generate predictions for the test data. The functional relationship between MaxP and PIN obtained from the cross-validated prediction set was applied to the test data MaxP and PIN was estimated. The PINCom's predictions were subsequently estimated by the estimated PIN and MaxPIN (Methods). Since the test data consists of the elements whose true classes are known, we didn't use double cross-validated prediction data for an assessment.

**Figure 10:**
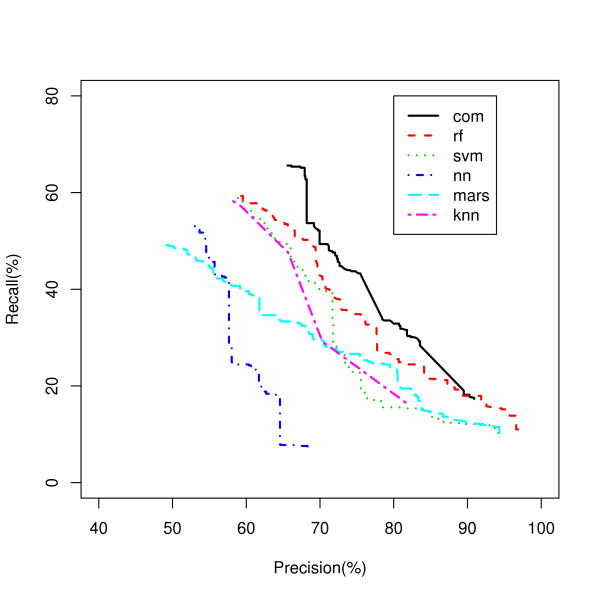
**PIN Combined Classifier Performance on Vowel Recognition Data**. Five classifiers classifying vowels are compared the PIN Combined Classifier. com - PIN Combined Classifier (PINCom); rf - random forest; mars - Multivariate Adaptive Regression Splines; svm - support vector machines; knn - K Nearest Neighbor; nn - neural network.

The Recall-Precision curves are presented in Figure 10. The overall precisions of the test set predictions are 53%, 59%, 49%, 59%, and 58% for nn, rf, mars, svm, and knn respectively. A similar range of precisions was reported in [15]. The PINCom (com) has the overall precision of 66%, which is 7% higher than the best classifier (rf or svm) and 17% higher than the worst classifier (mars). The best previously reported precision for this data is 61% [15]. The PINCom dominates not only on the overall recall but also on recalls of all the predictions at the precision levels up to 90% precision. A way to extend the range of precision value of the Combined Classifier to 100% is to use class specific precision index PIC similar to our previous example.

Recalls of the subsets with precision 70%, 80%, and 90% for all classifiers (Methods) are presented in Table 3. The overall precision of the vote-combining classifier Vote is surprisingly high, 63%, beating all the other classifiers except the PINCom. However, the recalls for the vote-combining method at the high precision levels of 70%, 80% and 90% are even lower than some of the individual classifiers.

**Table 3:** Recall Percentage of the individual classifiers and Combined Classifiers at different precision levels for Vowel Recognition Data

Precision	Overall (%)	70%	80%	90%
**rf**	59	42.7	26.2	18.0
**svm**	59	40.0	15.6	12.1
**nn**	53	4.1	2.0	0.0
**mars**	49	29.4	23.8	12.5
**knn**	58	29.2	16.4	0.0
**Vote**	63	39.0	17.3	0.0
**Stack 1**	57	36.3	27.0	3.6
**Stack 2**	62	43.0	22.0	6.0
**PINCom**	66	49.3	32.9	18.2

Stack 1 has the overall precision of 57%, slightly lower than the best individual classifier. The recalls at the precision levels of 70%, 80% and 90% are also lower than some of the individual classifiers. Stack 2 uses all the prediction probabilities and class predictions from 5 classifiers as level-1 data and a random forest as the level-1 classifier. The overall precision is 62%, higher than the best individual method and similar to Vote's but lower than PINCom's 66%. The recalls at the precision levels of 70%, 80% and 90% are comparable to the individual classifiers but are dominated by the PINCom.

## Discussion

We studied 5 classifiers for classifying genes to biological processes. Each classifier had significant advantages for certain biological classes under certain conditions, but no single model was optimal for all classifications. A way to combine the probability outcomes from all 5 classifiers into a superior model was developed by use of a performance measure (PIN) that normalized outcomes across the classifiers. We developed a Combined Classifier based on PIN measures for the 5 models yielding substantial improvements in performance overall and for individual classes of biological processes. The use of PIN in the Combined Classifier provided us the ability to select any desired precision across classes and models to yield the most genes classified. In Cell Cycle classification for example, the PIN Combined Classifier yielded a substantial number of genes classified with high confidence while all individual models yielded few or zero genes classified.

The individual classifiers are not built to classify data to multiple classes. For the genes with multiple functions, one could still use the classifiers by creating new classes for those genes and training the classifiers. However, the number of combinations of all multiple classes would be huge and the number of genes in each class might be too small to be useful in training. As the knowledge expands and enough information is gathered on those genes with multiple functions, we should be able to properly classify those genes to multiple classes using PINCom. For the sake of keeping the analysis straightforward, our study of the Combined Classifier focused on primary classifications only, leaving the classifications of secondary and tertiary functions for future research.

The analysis of contribution of each model to the Combined Classifier provided us insight into how each model contributed under various conditions of confidence. While a more detailed analysis on a class-by-class basis is needed, we can still see overall that the NN model, which showed overall the worst performance, is the best contributor. The results suggest that improvements in the Combined Classifier are possible in which the contributions of each model are further optimized.

## Conclusions

The advantages and implications of the Combined Classifier go well beyond models utilizing gene expression data and the classifiers used. Any classifier with estimated posterior probabilities can be used. There's no limitation to the number of models that can be combined. The use of PIN allows combining of models based on any type of data, such as classifiers based on protein sequence or protein-protein interaction data. This provides the foundation for integrating highly diverse and seemingly incongruent information into a single multi-class model with high performance. The advantages of the Combined Classifier also go beyond functional genomics and should also be apparent in broad fields of basic science, clinical science, and business, as the modeling of vowel recognition demonstrated.

## Authors' contributions

BW conceived of the study, developed, refined, tested, and applied the methods, and drafted the manuscript. DK formulated, developed, programmed, and refined the methods, and drafted the manuscript. All authors analyzed the data, read and approved the final manuscript.
